# Maximising the Potential Benefit of Living with Companion Dogs for Autistic Children and Their Families: A Mixed-Methods Survey of the Impact of a Novel ‘Family Dog Service’

**DOI:** 10.3390/ani15172492

**Published:** 2025-08-25

**Authors:** Emily Shoesmith, Heidi Stevens, Selina Gibsone, Cari Miles, Hannah Beal, Kelly Jennings, Elena Ratschen

**Affiliations:** 1 Department of Health Sciences, University of York, York YO10 5DD, UK; heidi.stevens@york.ac.uk; 2 Dogs for Good, The Frances Hay Centre, Blacklocks Hill, Banbury OX17 2BS, UK; selina.gibsone@dogsforgood.org (S.G.); cari.miles@dogsforgood.org (C.M.); hannah.beal@dogsforgood.org (H.B.); kelly.jennings@dogsforgood.org (K.J.)

**Keywords:** companion dogs, dogs, pets, autism, mental health, survey

## Abstract

**Background:** Assistance dogs can support children with autism by improving emotional regulation and social functioning, but access is limited. The Family Dog Service was developed to help families of autistic children gain similar benefits through companion dogs. It offers tailored support for selecting, training, and integrating a dog into the home. This study explored parent perspectives on the service and perceived impacts of companion dogs. **Methods:** A cross-sectional online survey was conducted among UK residents who attended Family Dog Service workshops. The survey included demographic data, mental health and wellbeing measures, and questions about human–animal interactions. Quantitative data were analysed descriptively; qualitative responses underwent thematic analysis. **Results:** Of 118 participants, 101 (85.6%) owned a dog, while 17 (14.4%) were considering acquisition. Most owners reported improvements in their child’s mood (75.2%; n = 76) and reduced anxiety-related behaviours (70.3%, n = 71) following dog acquisition. Nearly half (49.5%, n = 50) rated the child–dog relationship as ‘very’ or ‘extremely’ positive within the first month, increasing to 86.1% (n = 87) by the time of data collection. Families also reported enhanced dynamics and reduced caregiver stress. Despite some challenges, the service was valued for its autism-specific guidance and ongoing support. **Conclusions:** The Family Dog Service may offer a practical, accessible alternative to assistance dogs, supporting autistic children’s wellbeing and strengthening family relationships through positive interactions between children and their dogs.

## Simple Summary

Children with autism often benefit from specially trained assistance dogs, but these can be difficult to access. To address this problem, the ‘Family Dog Service’ was created to help families gain similar benefits by supporting families in choosing, training, and living with a regular companion dog. This study aimed to understand how families experienced the service and how having a dog affected their child and overall family life. An online survey was completed by 118 families in the United Kingdom who attended the service’s workshops. Most families already owned a dog, while others were planning to get one. Parents reported that their child’s mood improved and anxiety-related behaviours decreased after getting a dog. Many noticed a strong connection between their child and the dog within the first month, which grew stronger over time. Families also said that life at home became calmer and less stressful. Although some faced challenges with caring for the dog, the advice and continued support from the service were seen as helpful and autism-focused. These findings suggest that with appropriate support, companion dogs can improve the wellbeing of autistic children and their families, offering a more accessible option to improve quality of life and emotional health.

## 1. Introduction

Autism is a common neurodevelopmental condition that varies widely across individuals in terms of presentation and can affect numerous areas of life, including education, employment, independent living, and social participation [1]. It can be characterised by challenges in social communication and the display of restricted, repetitive behaviours [2]. It is well established that autism should be diagnosed as early as possible and that early intervention, tailored to individual needs, is important for achieving positive outcomes [3,4]. The central role of psychological and practical resources available to primary caregivers of autistic children has been highlighted to support both the child and the wider family [5].

Among various support strategies, the role of animals, especially dogs, in improving the wellbeing of children with autism has gained increasing attention [6,7,8,9,10]. Assistance dogs, specifically trained to support individuals with living with disabilities or long-term medical conditions [11], have been shown to improve emotional regulation, increase social engagement and community participation, and reduce anxiety in children with autism [7,10,12,13,14]. Moreover, it has been reported that assistance dogs can improve the quality of life of the wider family by fostering family cohesion and reducing caregiver stress [6,7,9,10]. However, access to such highly trained assistance dogs is limited due to strict eligibility criteria, long waiting lists, and substantial costs associated with specialist training [15].

Evidence suggests that companion dogs, despite usually lacking formal or highly specialised training focused on autism, might also have the potential to support the development of social and emotional skills [16], increase physical activity [16], and reduce anxiety [17] in autistic children. Despite these benefits, negative aspects related to dog ownership have also been described, for example relating to the time commitment and work required when training and caring for a dog [15,18]. For families with autistic children, managing the relationship between the child and the dog may also present unique challenges, such as interpreting the dog’s cues, ensuring consistent routines, and accommodating sensory sensitivities or communication differences [7,16,19,20]. To harness the full potential of a companion dog to benefit the lives of autistic children and their families, access to tailored training courses and support and guidance on how to manage the human–animal relationship might benefit both the family and the dog [21] and offer opportunities to maximise the potential of living with a companion dog.

Dogs for Good, a charity based in the United Kingdom (UK), have designed an innovative service, named the Family Dog Service, tailored to families with a child (or children) with autism, to provide parents the advice and long-term support required for choosing and training a dog to benefit the whole family. The workshops involved in the service aim to harness the full potential of a companion dog, while promoting a positive reinforcement approach and prioritising the welfare of the dog. Training based on the principles of positive reinforcement is reported as more beneficial and valuable for dog owners than other types of training [22] and more effective in producing well-trained and well-socialised companion dogs [23].

The Family Dog Service has recently been explored in a qualitative study, which involved interviews with 16 parents of children with autism who had engaged with the service [19]. The findings indicated that companion dog ownership provided emotional and physical benefits to children with autism and their families, similar to those reported for autism assistance dogs [19]. Although the findings do not imply companion dogs can substitute for the vital role of specially trained assistance dogs, it is likely the reported benefits may arise from the development of the human–dog bond, supported by the Family Dog Service. The aim of the current survey study was to corroborate and further explore parent experiences of the Family Dog Service and the perceived impact of human–animal interactions and relationships on children with autism and their families in a larger sample. Specifically, we investigated the following questions:

1. What are the key considerations and challenges for families with a child (or children) with autism when selecting a companion dog? (RQ1)
2. What is the perceived impact of the companion dog on children with autism and their wider family networks? (RQ2)
3. What are the experiences and perceptions of engaging with the Family Dog Service? (RQ3)

## 2. Materials and Methods

### 2.1. Study Design

A cross-sectional, retrospective survey.

### 2.2. Overview of the Family Dog Service

The Family Dog Service is described in detail elsewhere [19], but in summary, Dogs for Good (formerly Dogs for the Disabled) offers the Family Dog Service to support families of children with autism (ages 3–16), including those on the diagnostic pathway. The service offers workshops to families at every stage of dog ownership, including (1) during initial stages when families are exploring and researching how a dog could assist their child; (2) helping families select, introduce, and train a dog; and (3) offering guidance to families who already have a companion dog. Importantly, families select and train their own dogs, with the Family Dog Service offering support and advice, rather than conducting direct monitoring, assessments, or welfare checks. As a result, the characteristics and training approaches of the dogs involved will vary.

Launched in 2010, the Family Dog Service transitioned to online workshops in 2020 due to the COVID-19 pandemic. The workshops are pre-recorded and accessible via Microsoft SharePoint, with live follow-up sessions on Microsoft Teams for Q&A and group discussions. Families also receive lifetime support from the Family Dog Service team, access to a private Facebook group for advice, and additional resources like fact sheets and videos to complement the workshops.

### 2.3. Setting and Participants

The survey was conducted among parents who had engaged with the Family Dog Service, irrespective of dog ownership.

### 2.4. Recruitment and Procedures

The survey was hosted on Qualtrics (Qualtrics, Provo, UT, USA) and made available via the private Family Dog Service’s closed Facebook group. Access to this group is restricted to families who have either completed or are actively engaged in the Family Dog Service workshops. Members were invited to take part by following a survey link, which first directed them to a Participant Information Sheet outlining the purpose of the study. Participants provided informed consent electronically by checking a box before proceeding. To ensure eligibility, a screening question confirmed that participants had attended at least one Family Dog Service workshop, with those who had not being unable to continue. Survey responses were collected anonymously and stored securely on the University of York’s Qualtrics server.

The survey commenced on 30 May 2024 and ended on 30 September 2024. Ethical approval for the study was granted by the Health Sciences Research Ethics Committee at the University of York, UK, on 21 November 2023 (ref: HSRGC/2023/596/B).

### 2.5. Measures

To develop the survey, semi-structured interviews were conducted with a sub-sample of parents who had engaged with the Family Dog Service [19]. Based on this qualitative data and in collaboration with the Dogs for Good team, a bespoke questionnaire was generated (Supplementary Material S1) to enable collection of data most relevant to the research questions, as detailed below.

#### 2.5.1. Demographic Data

Demographic information was gathered about participants’ age (in bands), gender (male, female, or non-binary), ethnicity (e.g., White, Mixed, or Black), number of children, number of children with a diagnosis (or suspected diagnosis) of autism, and whether the child(ren) with autism had any comorbid (or suspected comorbid) diagnoses. Throughout this manuscript, we use either identity-first language (e.g., ‘autistic child’) or person-first language (e.g., ‘child with autism’), reflecting the terminology preferences expressed by the families and children who took part in this research.

#### 2.5.2. Dog Ownership

Participants were asked if they currently have a family dog supporting their child(ren) with autism. Those with more than one dog were asked to identify the dog primarily supporting their child(ren) and answer questions about that dog. Information was collected on the dog’s breed, how long they had owned the dog, and factors considered when selecting the breed (e.g., personality traits, size of dog, and sensory preferences). Information was also collected on whether participants experienced any challenges when acquiring their dog (e.g., long waiting lists or rejection due to autism/neurodiversity) or integrating their dog into the family unit (e.g., challenges associated with the development of the human–dog bond or concerns around training). Participants were asked to specify whether they had contacted Dogs for Good for support in relation to these challenges (yes/no), and if so, how helpful the team was in helping to mitigate these challenges on a 7-point Likert scale (1 = extremely unhelpful; 7 = extremely helpful).

#### 2.5.3. Perceived Impact of the Dog on the Child with Autism

If participants had more than one child with autism, they were asked to answer the following questions thinking about the child the family dog primarily supports. Participants were asked to specify what the main goals and expectations were for their family dog from eight pre-determined responses (e.g., to provide companionship, to promote physical activity, or to reduce anxiety-based behaviour), or to specify themselves. A 5-point Likert scale was provided for participants to indicate whether the dog had met their goals and expectations (1 = fallen short of all goals/expectations; 5 = exceeded goals/expectations).

Participants were asked to indicate their perceptions of the relationship between the family dog and child in the first four weeks of the dog’s arrival and at the present time of completing the survey on a 7-point Likert scale (1 = extremely negative; 7 = extremely positive). A question was also presented to ask whether the dog had a positive impact on specific domains based on the Lincoln Autism Pet Dog Impact Scale [15] (yes/no). These included 25 pre-determined responses (e.g., social skills, tolerance in changes in routine, verbal or non-verbal communication, and sleeping patterns), or to specify themselves.

#### 2.5.4. Perceived Impact of the Dog on the Wider Family

Participants were asked to indicate their perceptions of their relationship with the family dog in the first four weeks of the dog’s arrival and at the present time of completing the survey using a 7-point Likert scale (1 = extremely negative; 7 = extremely positive). A question was also presented to ask participants what impact their dog has had in specific domains (e.g., calm household, time available for respite, physical activity, and social support and companionship) on a 7-point Likert scale (1 = extremely negative; 7 = extremely positive).

#### 2.5.5. Experiences of the Family Dog Service

Participants were asked to indicate how they heard about the Family Dog Service and what factors influenced their decision to attend the workshops. Participants were then asked to specify the top three areas covered during the workshops they found most helpful from 23 pre-determined options (e.g., safety in the home and appropriate behaviour, understanding dog language and communication, the theory of how dogs learn, how to set up training sessions, and advanced taskwork). Additionally, participants were asked to specify how helpful and important they found core features of the service (e.g., interactive nature of the workshops, being in a group with other parents/carers, and access to lifetime support) using a 7-point Likert scale (1 = extremely unhelpful, unimportant; 7 = extremely helpful, extremely important). Participants were asked to briefly describe how the workshops compared to a conventional training course, and if they would recommend the service to others.

Participants were also asked whether they attended the workshops in person or online, whether they were happy with the mode of delivery (yes, partially; would have preferred a hybrid, no). A free-text, open-ended box was presented for participants to briefly describe why they were (or were not) happy with the mode of delivery.

Lastly, the survey included the option for participants to leave an open-ended, free-text comment to describe their experiences and perceptions of the Family Dog Service and the perceived impact of their family dog, where applicable. The item read: “If you have any further comments related to your experience with the Family Dog Service or the impact of your family dog on your family, please leave them here”.

### 2.6. Data Analysis

Descriptive summary statistics are presented for demographic variables and data relating to companion dog ownership, parent perceptions regarding considerations and challenges of dog ownership and the impact of their family dog, and experiences of the Family Dog Service.

Open-text responses were imported into NVivo 12 software (QSR International Pty Ltd. Version 12, Ottawa, ON, Canada). Thematic analysis [24] was conducted using an inductive approach, with codes and themes developed directly from participants’ accounts. To begin, one researcher immersed herself in the dataset by reading all comments and noting potential codes based on recurring ideas and language. These preliminary codes were then refined and grouped into broader categories, which were subsequently organised into potential themes. All relevant excerpts were collated within each theme. Two researchers independently examined the coding framework, theme structure, and illustrative quotations before reaching consensus.

## 3. Results

A total of 124 eligible participants consented to taking part in the study. Of those, six had answered no more than the first survey question and were removed for the purposes of data analysis, resulting in a final sample of 118 participants. A summary of participant characteristics is presented in Table 1.

Participants reported having a total of 251 children, of whom 69.7% (n = 175) had a confirmed or suspected diagnosis of autism. Of the 175 children with a diagnosis (or suspected diagnosis) of autism, 92 (52.6%) were male, 74 (42.3%) were female, five (2.9%) identified as non-binary/third gender, and four (2.2%) parents preferred not to specify. The mean age of children with autism at the time parents began the workshops was 9.63 years.

Of the 175 children with a diagnosis (or suspected diagnosis) of autism, 74.9% (n = 131) were reported to have a comorbid (or suspected comorbid) diagnosis. Commonly reported comorbidities included attention deficit hyperactivity disorder (ADHD) (48.1%; n = 63), learning disabilities (e.g., dyslexia or dyspraxia) (23.7%; n = 31), depression or anxiety (13.7%; n = 18), and obsessive–compulsive disorder (9.2%; n = 12).

The majority of participants (85.6%, n = 101) reported owning a companion dog at the time of data collection. Among these, 62.4% (n = 63) had owned their dog for over two years, 18.8% (n = 19) for one to two years, and 18.8% (n = 19) for less than one year. The most commonly reported breeds were Labradors (43.6%; n = 44) and Poodle and Cocker Spaniel crossbreeds (17.7%; n = 18). Seventeen participants (14.4%) did not yet own a dog but were actively considering dog acquisition and had engaged with the Family Dog Service specifically to support the decision-making process around selecting a suitable companion dog for their child and family context.

### 3.1. What Are the Key Considerations and Challenges for Families with a Child (or Children) with Autism When Selecting a Companion Dog? (RQ1)

Companion dog owners (n = 101) identified several factors they considered important when selecting a family dog. These factors included temperament and personality traits (90.1%; n = 91), size of the dog (76.2%; n = 77), trainability (71.3%; n = 72), sensory preferences such as coat type and length (49.5%; n = 50), dog gender (27.7%; n = 28), and coat colour (17.8%; n = 18). Additionally, 19 participants reported other considerations not pre-specified in the survey, such as the desire for a hypoallergenic breed (38.8%; n = 7), prior ownership experience with specific breeds (21.1%; n = 4), and consideration of the presence of other companion animals in the household (15.8%; n = 3).

Once a breed was selected, more than half of the participants (59.4%; n = 60) reported experiencing challenges during the process of purchasing or adopting their dog. Of these, challenges included financial costs associated with acquiring a dog (60.0%; n = 36), difficulties finding a reputable or ethical breeder (43.3%; n = 26), long waiting lists (36.7%; n = 22), rejection based on neurodiversity (10.0%; n = 6), and communication issues with breeders (8.3%; n = 5).

**Figure 1:**
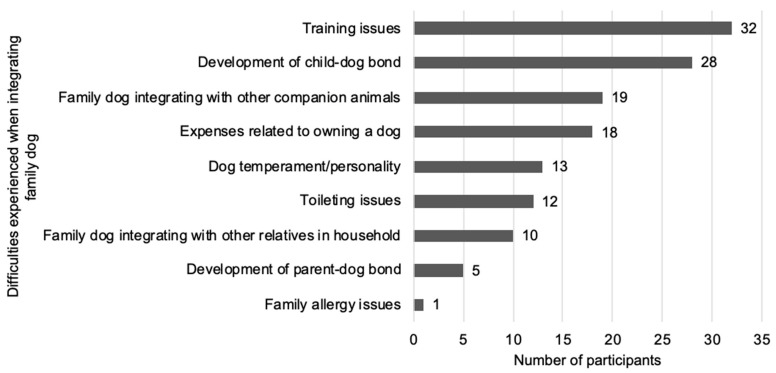
Difficulties experienced when integrating companion dogs into the family home.

In terms of integrating the dog into their home, over half of the participants reported that the process was ‘somewhat easy’ (21.8%; n = 22), ‘very easy’ (19.8%; n = 20), or ‘extremely easy’ (20.8%; n = 21). However, 3.0% (n = 3) found integration to be ‘extremely difficult’, 3.0% (n = 3) found it ‘very difficult’, and 23.8% (n = 24) found it ‘somewhat difficult’. A smaller proportion, 7.9% (n = 8), indicated that integration was ‘neither difficult nor easy’. The common challenges associated with integrating a dog into the home are summarised in Figure 1.

Seventy-seven participants reported that they contacted the Family Dog Service team in relation to challenges integrating their dog into the family home. Of these 77 participants, 70 (90.9%) reported the team were ‘somewhat’, ‘very’, or ‘extremely’ helpful in terms of mitigating these challenges.

### 3.2. What Is the Perceived Impact of the Companion Dog on Children with Autism and Their Wider Family Networks? (RQ2)

#### 3.2.1. Quantitative Data

Participants who currently owned a family dog (n = 101) were asked to identify their primary goals and expectations for acquiring a family dog. The most frequently cited goals included providing companionship for their child with autism (90.2%; n = 81), reducing anxiety-based behaviours (71.3%; n = 72), and improving their child’s mental health and wellbeing (71.3%; n = 72). Other common expectations included fostering a calming influence on family dynamics (56.4%; n = 57), promoting their child’s independence and sense of purpose (49.5%; n = 50), encouraging physical activity (41.6%; n = 42), enhancing their child’s social skills (39.6%; n = 40), and increasing their child’s access to the local community (37.6%; n = 38).

Within the first four weeks following the arrival of the companion dog, the majority of participants reported a positive relationship between the dog and their child (76.2%; n = 77). However, some participants initially described the relationship as negative (17.8%; n = 18). By the time of data collection, only two participants (2.0%) reported that the relationship remained negative (Table 2).

**Figure 2:**
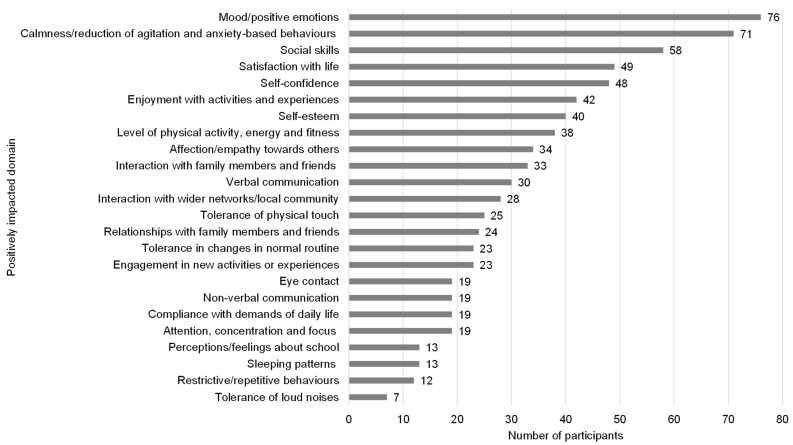
Perceptions of owners regarding the positive impact of their companion dog on their child with autism.

All participants indicated that their companion dog had a positive impact on at least one domain for their child with autism (Figure 2). The majority of participants reported improvements in their child’s mental health and mood (75.2%; n = 76) and an increase in calmness, with a reduction in agitation and anxiety-related behaviours (70.3%; n = 71). More than half of the participants also noted that their companion dog had positively influenced their child’s social skills (57.4%; n = 58). No participants indicated there had been no perceived benefits of their dog for their child with autism.

Within the first four weeks of the companion dog’s arrival, the majority of parents reported their own relationship with their companion dog was positive (89.1%, n = 90), whereas ten (9.9%) reported it was negative. However, at the point of data collection, only one participant (1.0%) reported their relationship remained negative (Table 3).

**Figure 3:**
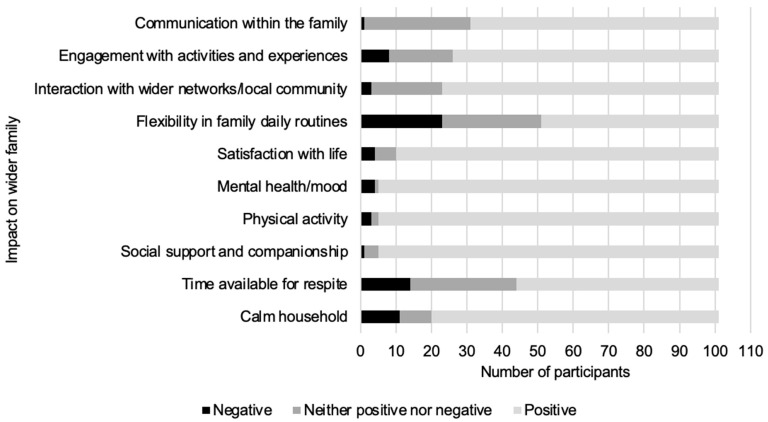
Perceptions of owners regarding the impact of their companion dog on their wider family.

The majority of participants indicated their companion dog had a positive impact on their wider family network in several domains (Figure 3). The domains most commonly rated as ‘somewhat positively’, ‘very positively’, or ‘extremely positively’ impacted by their companion dog included social support and companionship, promotion of physical activity, and improvement of mental health and mood (95.0%, n = 96). Domains most commonly rated as ‘extremely negatively’, ‘very negatively’, or ‘somewhat negatively’ impacted by their companion dog included flexibility in family daily routines (22.8%, n = 23) and time available for respite (13.9%, n = 11).

#### 3.2.2. Qualitative Data

Of the 118 participants, 86 provided a response to optional free-text items. The thematic analysis of free-text responses resulted in the identification of three main themes related to various aspects of the perceived impact of the family dog, including (1) the positive impact of dog ownership on children with autism, (2) the positive impact of dog ownership on the wider family, and (3) challenges and considerations associated with dog ownership for families with a child (or children) with autism. To illustrate themes, the free-text responses are presented as verbatim quotes below.

##### The Positive Impact of Dog Ownership on Children with Autism

Many participants expressed deep appreciation for their companion dog, frequently noting that the dog had exceeded expectations by providing support across several domains of their child’s life, such as reducing anxiety, improving emotional regulation, and facilitating smoother transitions and flexibility in daily activities and schools.

*“I used criteria to help choose the correct puppy for my sons needs but I cannot believe how well it turned out. [Dog’s name] helps with deep pressure, anxiety, mood, going out the house, helping my son with meltdowns, calms him, provides sensory relief. My son seeks comfort off him, he helps with school transitions, communication, distraction, car journeys. The way [dog’s name] helps, the list is endless.”*

*“My daughter has had to be supported by a crisis team, and she has said [dog’s name] calmness and cuddling her has been the main thing that has helped with her mood and emotional regulation.”*

*“I didn’t expect to have such flexibility in daily routines now my child is much more willing and open to change. I didn’t expect a four-legged creature to have such an impact on this.”*

Additionally, many participants reported that their dog facilitated improvements in their child’s social interaction and communication skills, extending beyond immediate family members to include interactions with extended family and friends.

*“Improvement in communication. Both my children find it easier to communicate with me when cuddling our dog or when out walking with her.”*

*“Our eldest son now TALKS!!! All because of developing communication skills with playing games with our dog!”*

*“My extended family all love her and often want to take her for walks or be with her, which then indirectly provides more social interaction for my children which improves their interaction.”*

*“He has benefitted our wider family both directly and indirectly. Everyone loves him and he brings a lot of joy but also our daughter is more willing to interact with them when he is around which has bought everyone closer together.”*

Participants consistently attributed these positive impacts to the dog’s unconditional love, affection, and companionship, which appeared to foster relationships characterised by the absence of judgement and a sense of acceptance.

*“[Dog’s name] ability to calm our child after meltdowns, it’s due to the empathetic bond between the two of them.”*

*“[Son’s name] was 9 when we got [dog’s name]. It was a very considered decision as we already had our hands full with extreme autistic behaviours, so attending workshops helped seal the deal. Sadly, [dog’s name] died when my son was 21. He grew up with her and she was his best (and only) friend. When his OCD got severe and we couldn’t get him out the house, she was the only reason he would go out. She helped him SO much over her lifetime—we are indebted to her. I could write a book about how she has helped us all and we are so sad she is no longer here.”*

##### The Positive Impact of Dog Ownership on the Wider Family

In addition to the direct benefits of the companion dog to the child with autism, parents stated that dog ownership resulted in engagement in animal-related activities (e.g., dog walking and dog training) which subsequently led to increased socialisation with friends or family. In this sense, their dog was perceived as a connection within their social network and also provided a sense of belonging in their community.

*“I had not realised the fact that [dog’s name] would create such a talking point, giving us all something we can talk about, inside and out of the house.”*

*“My husband has made new friends on his early morning dog walks, after 20 years of living here and making none.”*

*“Connecting to people in our village because of [dog’s name] was an unexpected bonus.”*

*“Our family adores him and he’s very popular with friends and neighbours who help walk or look after him occasionally; he has such a lovely temperament. One acquaintance has become a friend through our walking him together once a week; it’s been very good for her mental and physical health. And I’ve made friends through walking him that I would never have met otherwise.”*

Furthermore, many participants expressed that the presence of the dog led to positive shifts in familial dynamics. For example, participants frequently mentioned how the companion dog fostered closer family bonds and facilitated improved communication within the household.

*“Communication in the family has increased tenfold since introducing [dog’s name]. She acts as a buffer for difficult communications and melts everyone’s hearts which just brings so much joy into the household.”*

*“Unexpected benefits of how much the dog has bought the family together. I knew it would have a positive impact, but I didn’t realise the extent to how it’s changed our lives.”*

*“Our son is an only child so having a dog helped our son to think about our dog and her needs, this was a very positive shift for our family.”*

Some participants indicated that the presence of their dog alleviated their child’s dependency on parental support, offering them opportunity for additional respite, which was often perceived as an unexpected impact.

*“Our dog has lightened my daughter’s dependency on me. I didn’t expect to need our dog in this way, so it is a bonus.”*

*“It has given me the opportunity to walk the dog sometimes by myself, it’s respite I didn’t previously have.”*

Lastly, many participants reported that their companion dog had positive effects on individuals beyond the immediate household, such as extended family members and friends. Although these broader social impacts were not initially anticipated by participants, they were frequently noted and highly valued.

*“Close family love dogs and have benefited from the relationship with our dog. My father is currently in a nursing home and loves the dogs visits!”*

*“My mother has bipolar, and we take the dog round there sometimes to her house and it brightens her day when she is down.”*

*“She is taken for regular walks by a friend who finds it aids their mental health. She is also helpful to the mood of elderly relations if we take her to visit.”*

*“Everyone who met him had positive experiences with him particularly other young extended family members (cousins) who also have autism. He has a very calming influence.”*

##### Challenges and Considerations Associated with Dog Ownership for Families with a Child (or Children) with Autism

Some participants discussed the difficulties of incorporating a new dog into their family and adapting to a different lifestyle and routine. This shift appeared to negatively impact some parents, who highlighted the responsibility of caring for a dog was more demanding and time-consuming than they had originally anticipated. This prompted parents to emphasise the importance of considering the potential challenges associated with effectively caring for a dog prior to acquisition, particularly in contexts when additional demands were already placed on the family.

*“We did not anticipate how much extra care would need to be taken around the dog-child interaction which makes caring more tiring and respite harder.”*

*“Acknowledgement that any dog complicates your life, and if you already have a lot of demands, it may not be worth it.”*

*“He is not a temperament that means he can come to work with me, which has had a really negative impact, as I am having to work from home a lot. Because of my son’s needs, this means I don’t get to talk to anyone who can hold a conversation in a whole day. Our dog is training really well, but the impact on mental health for us all has taken a significant toll in a family who is already stretched due to SEN needs. We all love the dog and will work hard with him, and I do have faith in 18 months, we’ll be in a very different situation, but if I could go back in time, I would not do it. I would definitely be very cautious in advising other families with all the restrictions and needs that autism brings to get a dog.”*

Additionally, some participants spoke about the sensory impacts associated with the dog, indicating certain stimuli had introduced unforeseen challenges for both the child and other relatives.

*“Calm environment is the only negative. Our previous Labrador never barked. Our current one does (a lot), and both my spouse and ASD child are very triggered by the barking.”*

*“My son has struggles with the drool and the moulting and tends to not like the way our dog feels. This has impacted the way their relationship has gone. He still loves her but doesn’t cope with certain elements well.”*

Conversely, some parents indicated the time-consuming nature of dog ownership, especially during the initial stages, was worthwhile due to the positive outcomes for both the child with autism and the wider family unit.

*“I did find it hard initially having a dog—growing up with one hadn’t prepared me at all for the restrictions/arrangements necessary in terms of going out and having to walk him daily/twice daily but very used to it now and it’s all been extremely positive.”*

*“I was surprised by the amount of stress owning a dog would involve, that said, we would not be without him now!”*

### 3.3. What Are the Experiences and Perceptions of Engaging with the Family Dog Service? (RQ3)

#### 3.3.1. Quantitative Data

##### Factors Influencing Decisions to Attend the Family Dog Service

Participants primarily engaged with the Family Dog Service to seek assistance in training their companion dog to support their child(ren) with autism, with 78.0% (n = 92) citing this as their main reason for participation. Other notable reasons for engagement included access to lifetime support and advice (53.4%; n = 63), the perception that the service was more suitable for family needs compared to conventional dog training courses (46.6%; n = 55), child(ren) not meeting the criteria for an assistance dog (e.g., due to the child’s age) (31.4%; n = 37), and long waiting lists for assistance dogs (24.6%; n = 29), or the closure of these lists (15.3%; n = 18).

##### Mode of Delivery

Prior to the transition to remote delivery in 2020 due to the COVID-19 pandemic, 44 participants (37.3%) attended the workshops in person. Among these, the majority (93.2%; n = 41) reported being completely satisfied with the in-person delivery format, while a small number of participants (6.8%; n = 3) indicated that a hybrid delivery approach would have been beneficial. No participants expressed dissatisfaction with the in-person delivery method.

Seventy-four participants (62.7%) attended the workshops online. Among these, 74% (n = 55) were completely satisfied with the remote delivery format. However, 20.3% (n = 15) of participants suggested that some in-person opportunities would have enhanced their experience, while 5.4% (n = 4) indicated a preference for attending entirely in-person workshops.

##### Family Dog Service Features

Regardless of mode of delivery, the majority of workshop features were rated as ‘somewhat’, ‘very’, or ‘extremely’ helpful and important (Table 4). Highly rated features included the workshops themselves (either in person or remotely), access to lifetime support and advice, and the interactive nature of the workshops. Access to a private Facebook group for peer support was the feature most likely to be rated as unhelpful or unimportant.

##### Workshop Content

When participants were asked to select the top three areas covered during the workshops that they found most helpful, the most commonly selected topic was understanding dog language and communication (44.9%; n = 53), how to build the relationship between the dog and the child (43.2%; n = 51), how the dog can help the child (40.2%; n = 47), and advanced taskwork (e.g., head rest, body rest, nose nudge, and button push) (27.1%; n = 32). The content least likely to be selected included information on dog health (e.g., how to perform a basic health check) and considerations around grooming (0.8%; n = 1, respectively). Table 5 presents all workshop content and how many participants chose each topic within their top three selections.

#### 3.3.2. Qualitative Data

The thematic analysis of free-text responses resulted in the identification of two main themes related to experiences of the Family Dog Service, including (1) enhanced experience beyond conventional dog training and (2) perceptions on mode of delivery. To illustrate themes, the free-text responses are presented as verbatim quotes below.

##### Enhanced Experience Beyond Conventional Dog Training

The majority of participants expressed the value of the dual focus on dog behaviour/welfare and autism-specific information and support that went well beyond that of conventional training courses. Participants highlighted this specialised understanding allowed for guidance and support that was relevant and effective in meeting their unique needs.

*“The specific focus because of the understanding of both dogs and ASD behaviours meant advice more applicable and therefore more useful.”*

*“Much more focused on my son’s needs as well as supporting the dog, bringing both needs together in one place and providing continuous support for an autistic family that is not a static condition. Things continuously change and evolve, and the support changed and evolved with it as needed. Wouldn’t have had this in any other course.”*

*“Very tailored towards our family needs, putting my daughter first as well as the dog. We had attended conventional training courses previously and it was not helpful for our needs as they didn’t account for nuances and continuous changes in the family.”*

*“It was light at the end of a very dark tunnel to stumble across the Family Dog Service, I had no idea I could be empowered to provide my daughter with a dog she so desperately needed to manage everyday life. It’s so difficult with long lead-times to have a training dog provided, we couldn’t wait. The service has enabled me to change my daughter’s life for the better.”*

Specifically, many participants cited how the Family Dog Service had facilitated the development of the child–dog bond, a valuable and unique component of the service that participants noted would not be involved in conventional dog training courses.

*“It focused more on positive reinforcement and positive relationship building with my son and my dog more so than it did when I started attending a normal training course that I didn’t end up pursuing.”*

*“We also attended puppy training classes for socialisation and basic training, lead walk training to further his socialisation around other dogs […] but this is encouraged by the Family Dog Service anyway. However, no conventional training class could possibly have supported with individual needs of my son and our puppy and how to support that. I learned so much from the service to help those critical first 6 months together in our particularly challenging single parent family. This helped to build a relationship between my son and our dog that I didn’t know was possible. The team’s knowledge of AUTISM, tried and tested things to try at a time when I was shattered and on the verge of giving up the puppy, were completely essential to me.”*

Additionally, participants commonly expressed appreciation for the teams’ specialised knowledge and sensitivity to autism-related needs, highlighting how this understanding fostered a more supportive and inclusive environment, attuned to the unique challenges faced by families living with autism.

*“I really got the opportunity to talk about whether getting a dog was right for us and to weigh up all the options, the benefits and potential issues that we may meet. There is no other forum that I am aware of that we could discuss this with. Particularly with a team who are so knowledgeable about dogs and families with autism.”*

*“It’s impossible to find a course that combines invaluable knowledge, experience and support for both your child with neurodiversity, your dog and even the family as a whole. It’s the first time I felt a sense of relief knowing that I had such a knowledgeable team behind me.”*

*“Approaching from an autism perspective makes these workshops so unique and so helpful. As a parent it feels accessible and as if the barriers experienced elsewhere are removed. It makes such a difference when there is an understanding of autism and disability, it becomes welcoming and encouraging as opposed to draining and isolating.”*

##### Perceptions on Mode of Delivery

As the survey was open to anyone who had engaged with the Family Dog Service, participants varied in terms of their mode of engagement, with some attending in person and others participating remotely. This variation resulted from the service’s transition from in-person to online delivery in September 2020 due to COVID-19. The majority of participants who had engaged with the service since 2020 reported satisfaction with remote delivery due to accessibility and convenience.

*“I liked being able to watch videos in my own time and that they were broken down into manageable chunks i.e., 15 mins long. Then I enjoyed asking questions and discussing together as a group on the live meetings.”*

*“As a single mum it fit into my schedule perfectly while still being able to access invaluable information in a group of like-minded people.”*

*“I am not local to Dogs for Good; it would have been too much commitment if in-person. It would have made this inaccessible especially as my daughter has separation anxiety.”*

However, some participants suggested they would have preferred some in-person opportunities, though they frequently acknowledged that travel to such workshops could present a barrier.

*“It would have been nice to have the option for in-person work but I know this was not possible during the time (COVID). This did not affect the delivery of the service in any way though as it has been a god send to us and the family.”*

*“I found it easy to access and it was during COVID times so online was the only option. It would be good to do follow ups afterwards in person now it’s safe to do so. The in person events have usually been too far to travel though.”*

For those who attended in person prior to the transition online, most participants expressed how beneficial it was to see ‘live’ demonstrations and be in the same physical space as the dogs and the Dogs for Good team.

*“I loved the relaxed friendly atmosphere and to see what these dogs can achieve through practical demonstration. I liked being in the same space as the dogs, it made it easier to understand dog body language.”*

*“Doing the in person workshop really help to visually see and understand training elements from the demonstration dog. Was nice to be able to wander and chat to other family’s about their experiences and share ideas as everyone was at different stages of their journey to support their child with a dog. We could also much more easily ask the course delivery team individual questions.”*

Despite these benefits, the location of the workshops and required travel was often cited as a barrier, indicating a hybrid approach may be beneficial.

*“When I attended there were only in person workshops available. We had to travel a long distance to attend the nearest workshop due to the geographical coverage at the time. My husband and I attended separately so one of us was available for the school run, but we would have attended together if we had lived nearer. We were very motivated to access the training, and it was even better than we had hoped. I found the in person workshops great back then. However, as my son is high risk for COVID, I would now welcome online delivery.”*

## 4. Discussion

This cross-sectional survey study offers important insights into the potential role of companion dogs in supporting the wellbeing of children with autism and their families, particularly when acquisition and integration are guided by structured, autism-focused support. Following the engagement with the Family Dog Service and the introduction of a companion dog, families commonly reported improvements in child wellbeing, reductions in anxiety-related behaviours, and enhanced family functioning. These findings suggest that tailored programmes such as the Family Dog Service might play a critical role in enabling families to optimise the potential of companion dogs in a more accessible, flexible, and family-led manner. Importantly, the provision of ongoing, individualised support offered by the Family Dog Service assisted families in navigating the often complex and demanding processes of selecting an appropriate dog, managing the transition into the home, and fostering a sustainable, positive human–animal relationship. While over half of the participants reported encountering challenges during acquisition or integration phases, many indicated that these difficulties were addressed through communication with the Family Dog Service, highlighting the important role of accessible, responsive support in helping families to overcome barriers and successfully integrate a companion dog into their lives.

### 4.1. Impact of the Companion Dog on Children with Autism and Their Wider Family Networks

Most parents reported their family dog had a positive impact on their child’s mental health and mood, and that their dog contributed to a reduction in agitation and anxiety-related behaviours. The existing literature has examined the mechanisms by which companion animals may influence mental health and wellbeing, with one prominent framework emphasising the role of social support. The ‘buffering hypothesis’ suggests that the protective effects of social support are particularly evident in the presence of stressors [25], and studies have shown that interaction with animals can alleviate the anxiety-inducing effects of stressful situations [26,27]. This suggests that direct interaction with an animal, and the emotional feedback they provide, may play a role in emotional regulation. These findings align with our current results, highlighting how the family dog is able to offer unique emotional and social support to the child to improve positive affect and reduce anxiety. The dog’s ability to engage with the child in a reciprocal, non-verbal manner may help to enhance emotional resilience, especially when emotional regulation is often a significant challenge in autism [28].

In addition to direct benefits reported for children with autism, the current findings also highlight the positive impact of the companion dog on the wider family network, a result that aligns with previous research [7,9,10,29,30]. The existing literature consistently demonstrates that parents of children with autism may experience elevated levels of stress and reduced quality of life [31,32,33], factors that are often linked to the severity of their child’s symptomology [34,35]. For example, adherence to rigid routines and the anticipation and management of anxiety-related behaviours can pose significant daily challenges to parents and may negatively impact parent–child interactions, which subsequently contribute to overall family stress [36]. Therefore, it is likely that the benefits of companion dogs for children, including reductions in anxiety and improvements in mood and prosocial behaviours, may extend to the wider family network. These positive outcomes for the child could help to alleviate some of the sources of parental stress and anxiety, thereby enhancing the overall quality of life for the entire family. This is consistent with previous research that suggests stress associated with parenting a child with autism tends to decrease over time, and these reductions may be facilitated with the acquisition of a companion dog [29].

While the findings do not imply companion dogs can replace the important role of specialist trained assistance dogs, the benefits reported in this study align with those seen with autism assistance dog placements, such as improved emotional regulation, community participation and social skills [7,10,12,13,14,37], as well as enhancing wider family wellbeing [7,9,10]. The current study, along with the existing literature on the impact of companion dogs in families with autism [19], suggests these positive outcomes may not be solely due to the specialised training of the assistance dog, but they may occur due to the presence of the dog and the bond that develops between the child, the dog, and the overall family. While this relationship evolves over time, participants in the current study emphasised the vital role the Family Dog Service played in laying the foundation for a healthy, supportive relationship between the dog and the child.

### 4.2. Key Considerations and Challenges in Companion Dog Ownership

While companion dogs may offer benefits for families living with autism, some participants reported that the initial demands of dog ownership were more burdensome than anticipated, with negative implications for both their mental health and family dynamics. This finding aligns with existing research that highlights the dual nature of companion dog ownership, where potential benefits can be accompanied by certain challenges. Although companion animals are often associated with positive outcomes such as reduction in anxiety and increased emotional support for families with autism [17,30], studies also indicate that dog ownership can introduce new stressors, particularly when families are already managing multiple caregiving responsibilities [38]. For example, at the stage of acquisition, there are multiple factors to consider (e.g., personality, size, and sensory preferences), as matching a dog’s characteristics to the family’s needs is critical for fostering a positive relationship between the dog and the child [21]. Additionally, at the stage of integration, the time-consuming nature of dog care, as well as issues related to training and behavioural management, may exacerbate stress for families who are already under significant strain [39]. These findings suggest that, for some families, particularly those with limited resources or pre-existing stressors, the perceived benefits of companion dog ownership may not always outweigh the challenges.

Importantly, the emotional and practical readiness of families for dog ownership must be carefully considered prior to acquisition. It is possible that the full benefits of companion dog ownership may only become particularly prominent after the dog has successfully integrated into the family unit. During these initial stages, families may experience an increase in stress levels as they navigate the challenges associated with this transition. This appeared to be reflected in participants’ perceptions of their relationship with their dog, which shifted over time, from the initial four weeks following the dog’s arrival to the point of data collection. Initially, 10 participants reported a negative relationship, with only one continuing to do so at the time of data collection. Similarly, 18 parents initially viewed their child’s relationship with the dog as negative, but only two maintained this view at the point of data collection. Qualitative responses further supported this trend, with several parents describing the challenges and time-consuming nature of incorporating a new dog into their family life. However, the Family Dog Service appears to play an important role in this process by offering comprehensive pre-acquisition guidance, helping families to develop realistic expectations, and providing tailored support specifically for families living with autism, ensuring families are prepared to navigate both the benefits and challenges of dog ownership.

### 4.3. Limitations

Several limitations must be considered when interpreting the findings from this study. Firstly, the sample size was small and potentially limits generalisability to our study population. Notably, there was an over-representation of female participants. This gender imbalance may bias the findings as the experiences and perspectives of females may differ from those of males, particularly in the context of family dynamics and caregiving roles. However, such bias is common in the field of human–animal interaction research [40]. Additionally, in childhood neurodevelopmental research, mothers are the primary reporters, and fathers are largely under-represented [41]. It is important for future research to ensure approaches are taken to facilitate the recruitment of male caregivers. Secondly, the nature of the survey invites self-selection bias, as participants who were more willing to engage in the survey may have had more positive experiences about their family dog or the Family Dog Service. This could lead to an over-representation of individuals with favourable views, thereby impacting the generalisability to the wider cohort.

We also recognise that parental perceptions during the first four weeks of dog ownership may reflect a period of adjustment. This is why we used this timeframe as the initial data point to capture early relationship development, while also asking parents to reflect on the relationship at the present time, representing the longest period of ownership for each family. Although ownership length varied across the sample (with 19 participants (19.8%) owning their dog for less than one year), for most families the ‘present time’ reflections captured relationships following more than one year of dog acquisition. Nevertheless, a structured, longer-term follow-up at 6 months or 1 year post-integration would be valuable to ensure consistent data collection across families and to provide a fuller understanding of the long-term benefits and challenges of companion dog ownership.

## 5. Conclusions

This study highlights the valuable role that companion dogs may play in supporting autistic children and their families, particularly when integration is facilitated through structured, autism-specific guidance. While companion dogs are not a substitute for highly trained (and often unavailable) assistance dogs, living with a companion dog may provide accessible and meaningful benefits. These benefits may include improvements in emotional regulation, reductions in anxiety, enhanced social functioning, and strengthened family cohesion—outcomes which appear to be maximised when families receive tailored support in the selection, training, and relationship-building processes involved in introducing a companion dog into the home. The Family Dog Service offers a promising model for delivering such support, equipping families with the knowledge, skills, and ongoing guidance needed to foster a positive human–animal relationship and to navigate the complexities of dog ownership in the context of autism. While challenges related to acquiring and integrating a companion dog were frequently noted, the findings suggest that these can be addressed through individualised and responsive support. Overall, these results highlight the potential of well-supported companion dog ownership as part of a broader, family-centred approach to enhancing the wellbeing of autistic children and strengthening family life.

## Figures and Tables

**Table 1:** Participant characteristics.

Characteristics		% (n)
Gender	Female	94.1 (111)
	Male	5.9 (7)
	Non-binary	0 (0)
	Prefer not to say	0 (0)
Age (years)	25–34 years	1.7 (2)
	35–44 years	35.6 (42)
	45–54 years	44.9 (53)
	55–64 years	17.8 (21)
Ethnicity	White	95.8 (113)
	Asian/Asian British	1.7 (2)
	Black/African/Caribbean/Black British	1.7 (2)
	Prefer not to say	0.8 (1)
Cohabitation	With partner/spouse	72.0 (85)
	With children <18 years	81.4 (96)
	With other adults 18+ years	26.3 (31)
Number of children	1	26.3 (31)
	2	44.1 (52)
	3	23.7 (28)
	4	2.5 (3)
	5	3.4 (4)
Number of children diagnosed (or suspected diagnosis) of autism	1	64.4 (76)
	2	27.1 (32)
	3	5.9 (7)
	4	0.8 (1)
	5	1.7 (2)
Current companion dog ownership	Yes	85.6 (101)
	No	14.4 (17)

**Table 2:** Participant perceptions of the relationship between their child with autism and companion dog.

	Relationship in the First Four Weeks of Dog’s Arrival % (n)	Relationship at Present Time % (n)
Extremely negative	3.0 (3)	0 (0)
Very negative	2.0 (2)	1.0 (1)
Somewhat negative	12.9 (13)	1.0 (1)
Neither positive nor negative	5.9 (6)	1.0 (1)
Somewhat positive	26.7 (27)	10.9 (11)
Very positive	22.8 (23)	28.7 (29)
Extremely positive	26.7 (27)	57.4 (58)

**Table 3:** Participant perceptions of their relationship with their companion dog.

	Relationship in the First Four Weeks of Dog’s Arrival % (n)	Relationship at Present Time % (n)
Extremely negative	2.0 (2)	0 (0)
Very negative	2.0 (2)	0 (0)
Somewhat negative	5.9 (6)	1.0 (1)
Neither positive nor negative	1.0 (1)	0 (0)
Somewhat positive	13.9 (14)	1.0 (1)
Very positive	26.7 (27)	17.8 (18)
Extremely positive	48.5 (49)	80.2 (81)

**Table 4:** Workshop features rated as helpful and important.

Remote Delivery (n = 74)		
Feature	Helpful % (n)	Important % (n)
Workshop Lives (on Teams)	93.2 (69)	93.2 (69)
Access to lifetime support and advice	91.9 (68)	94.6 (70)
Interactive nature of Workshop Lives	86.5 (64)	90.5 (67)
Being in a group with other parents/carers	83.8 (62)	87.8 (65)
Access to pre-recorded workshop content	89.2 (66)	90.5 (67)
Access to handouts/resources	86.5 (64)	87.8 (65)
Access to videos from workshops	90.5 (67)	90.5 (67)
Access to private Facebook group	83.8 (62)	77.0 (57)
**In-Person Delivery (n = 44)**		
**Feature**	**Helpful** % (N)	**Important** % (N)
In-person workshop presentations	90.9 (40)	95.5 (42)
Access to lifetime support and advice	88.6 (39)	93.2 (41)
Interactive nature of in-person workshops	90.9 (40)	95.5 (42)
Being in a group with other parents/carers	81.8 (36)	86.4 (38)
Watching dog training and demonstrations	88.6 (39)	95.5 (42)
Access to handouts/resources	86.4 (38)	84.1 (37)
Access to videos from workshops	77.3 (34)	84.1 (37)
Access to private Facebook group	61.4 (27)	61.4 (27)

**Table 5:** Workshop content selected as most helpful by participants.

Workshop	Workshop Content	% (n)
Introduction	Selecting the right dog	1.7 (2)
Workshop One	Looking after your dog (including meeting their needs)	24.6 (29)
	Food, nutrition, and diet	1.7 (2)
	Health (including how to perform a basic health check)	0.8 (1)
	Considerations around grooming	0.8 (1)
	Safety in the home and appropriate behaviour	16.9 (20)
	Positive mental experiences for the dog in their experience of growing and learning	20.3 (24)
	Laws that apply to you as dog owners	11.9 (14)
	Family dynamics	4.2 (5)
	How to help build the relationship between your dog and your child	43.2 (51)
	Things to consider in the household that may prevent or hinder the relationship	1.7 (2)
	Ways to help your child build the valuable relationship	21.2 (25)
Workshop Two	How we can help our dogs in the home to make sure they are safe and comfortable	5.9 (7)
	Understanding dog language and communication	44.9 (53)
	What to do with your dog’s communication	4.2 (5)
	How your dog can help your child	40.2 (47)
	The theory of how dogs learn	8.5 (10)
	How to encourage wanted behaviour	4.2 (5)
	Impact of negative methods on the relationship with your dog	1.7 (2)
	What to do about unwanted behaviour	5.1 (6)
Workshop Three	How to set up a training session	1.7 (2)
	Different styles of training tools to help you and your dog	7.6 (9)
	Advanced taskwork (head rest, body rest, nose nudge, and button push)	27.1 (32)

## Data Availability

All relevant data are uploaded to the OSF repository and available via the following URL: https://osf.io/6m3rh/ (accessed on 14 March 2025).

## Supplementary Materials

The following supporting information can be downloaded at: https://www.mdpi.com/article/10.3390/ani15172492/s1, Supplementary Material S1: Questionnaire items.
