# Supplementary material for: Maximising the Potential Benefit of Living with Companion Dogs for Autistic Children and Their Families: A Mixed-Methods Survey of the Impact of a Novel ‘Family Dog Service’
Source: Animals (Basel). 2025 Aug 25;15(17):2492. doi: 10.3390/ani15172492 (PMC12427255; doi:10.3390/ani15172492)
Supplement: Supplementary file 1 [file animals-15-02492-s001.zip › animals-3832741-supplementary.pdf]

Supplementary Material 1. Questionnaire items

**00**	**Have you participated (or are currently participating in) the Family Dog Service workshops?**	Yes	
		No	**_END**
		**SECTION A: Demographics**	
**1a**	**Which of the following best describes how you think of yourself? I think of myself as…**	Male	
		Female	
		In another way	
		Prefer not to say	
**1b**	**Please tell us how old you are.**	18-24 years	
		25-34 years	
		35-44 years	
		45-54 years	
		55-64 years	
		65-70 years	
		Over 70 years	
**1c**	**Where do you live?**	Large city (100,000+ population)	
		Small city or town	
		Village or countryside	
**1d**	**What is your ethnic group?**	White (any white background)	
		Mixed/multiple ethnic group	
		Asian/Asian British	
		Black/African/Caribbean/Black British	
		Chinese	
		Arab	
		Other ethnic	
		Prefer not to say	
**1e**	**Who do you live with in your household?** *(Mark all that apply)*. **I live…**	With my partner/spouse	
		With children < 18 years old	
		With adults 18-70 years old	
		**SECTION B: About your child(ren)**	
**2a**	**How many children do you have?**	Rolling 1 – 5+	
**2b**	**How many children in your family have been diagnosed (or have a suspected diagnosis) of autism?**	1	**Go to 2c**
		2	**Go to 2d**
		3	**Go to 2d**
		4+	**Go to 2d**
**2c**	**Does your child with autism have any comorbid (or suspected comorbid) diagnoses?**	[Free-text]	

	Comorbidity defined as more than one diagnosis occurring at the same time (e.g., autism & ADHD)		
**2d**	**Do your children with autism have any comorbid (or suspected comorbid) diagnoses?**<br><br>*Comorbidity defined as more than one diagnosis occurring at the same time (e.g., autism & ADHD)*		
**SECTION C: Your family dog**<br><br>**Please answer the following questions thinking about the family dog that primarily supports your child(ren) with autism. If you have more than one dog that supports your child(ren) with autism, please respond to these questions with only one dog in mind.**			
**3**	**Do you currently have a family dog to support your child with autism?**	Yes	
		No	**SKIP**
**3a**	**How long have you had your family dog to support your child with autism?**	Less than 6 months	
		6 months – 1 year	
		1 – 2 years	
		2 – 3 years	
		3 years +	
**4**	**What breed is your family dog?**	[Free-text]	
**4a**	**Where did you buy/adopt your dog from?**	From a breeder	
		Rescue centre	
		Through a friend/relative	
		Bred dog ourselves	
		Other (please specify)	
**4b**	**What factors were important to you when selecting the breed of your family dog?** *Select all that apply.*	Temperament/personality traits	
		Size of dog	
		Sensory preferences/needs (e.g., type/length of coat)	
		Colour	
		Trainability	

		Other (please specify)	
**5**	**Did you seek advice when selecting your family dog from any of the following?**	Dogs for Good	
		Family member/friend	
		Internet searches	
		Breeders	
		Local dog trainer	
		Other animal charity	
		I did not seek advice from others	
		Other (please specify)	
**6**	**Did you experience any difficulties when buying/adopting your dog?** *Please select all that apply.*	Long waiting lists	
		Difficulties with communication from breeders	
		Finding a reputable/ethical breeder	
		Difficulties finding right breed for us	
		Expense of buying a dog	
		Rejection due to autism/neurodiversity	
		Adoption criteria from dog rehoming organisations	
		I did not experience any difficulties buying/adopting my dog	
		Other (please specify)	
**7**	**After getting your dog, how difficult did you find it overall to integrate your dog into your family unit?**	Likert scale:<br>1 = Extremely difficult<br>2 = Very difficult<br>3 = Somewhat difficult<br>4 = Neither difficult nor easy<br>5 = Somewhat easy<br>6 = Very easy<br>7 = Extremely easy	
**8**	**Did you experience any challenges when integrating your dog into the family unit?**	Challenges associated with the development of the human-dog bond for my child	
		Challenges associated with the development of the human-dog bond for me	
		Toileting issues	
		Challenges with dog temperament	
		Concerns around training	
		Family dog integrating with other pets in the household	
		Family dog integrating with other family members in the household (e.g., siblings, spouse)	
		Family allergies to family dog	
		Expenses related to owning family dog	
		Other (please specify)	
		None	**SKIP**
**8a**	**Did you contact Dogs for Good for any**	Yes	
		No	**SKIP**

	**support in relation to these challenges?**		
**8b**	**How helpful did you find Dogs for Good in helping to mitigate these challenges?**	Likert scale:<br>1 = Extremely unhelpful<br>2 = Very unhelpful<br>3 = Somewhat unhelpful<br>4 = Neither helpful nor unhelpful<br>5 = Somewhat helpful<br>6 = Very helpful<br>7 = Extremely helpful	
		**SECTION D: Family Dog Service**	
**9**	**How did you hear about the Family Dog Service?**	Contacted Dogs for Good for alternate reason (e.g., assistance dogs) and was informed about the service	
		Dogs for Good Facebook group	
		Other social media	
		Internet search	
		Recommendation from friend/family	
		Flyers/advertisements	
		Other (please specify)	
**10**	**What factors influenced your decision to attend the Family Dog workshops?** *Please select all that apply.*	Support for training our family dog to support our child(ren) with AUTISM	
		Waiting lists for assistance dogs were too long	
		Waiting lists for assistance dogs were closed	
		My child(ren) with AUTISM did not meet the criteria for an assistance dog (e.g., child's age)	
		Access to lifetime support and advice was appealing	
		Opportunity to learn how to train our dog ourselves	
		The workshops appeared to fit our needs more than conventional training courses	
		Helping to make a decision whether a dog was the right option for our family	
		Other (please specify)	
**11**	**What year did you begin the workshops?**	Rolling year	
**12**	**Which workshops have you attended?** *Please select all that apply.*	Introduction	**SKIP**
		WS1	
		WS2	
		WS3)	
**13**	**Please select the top 3 areas covered during the workshops that you found most helpful.**	*(Intro)* Selecting the right dog	
		(WS1) Looking after your dog (including meeting their needs)	
		Food, nutrition and diet	
		Health (including how to perform a basic health check)	

		Considerations around grooming	
		Safety in the home and appropriate behaviour	
		Positive mental experiences for the dog in their experience of growing and learning	
		Laws that apply to you as dog owners	
		Family dynamics	
		How to help build the relationship between your dog and your child	
		Things to consider in the household that may prevent or hinder the relationship	
		Ways to help your child build the valuable relationship	
		(WS2) How we can help our dogs in the home to make sure they are safe and comfortable	
		Understanding dog language and communication	
		What to do with your dog's communication	
		How your dog can help your child	
		The theory of how dogs learn	
		How to encourage wanted behaviour	
		Impact of negative methods on the relationship with your dog	
		What to do about unwanted behaviour	
		(WS3) How to set up a training session	
		Different styles of training tools to help you and your dog	
		Advanced taskwork (head rest, body rest, nose nudge, button push)	
**13a**	**Please briefly describe why you chose these areas as your top 3.**	[Free-text]	
**14**	**Did you attend the workshops in-person or online?**	In-person	**SKIP**
		Online	
**ONLINE WORKSHOPS ONLY**			
**15**	**How helpful have you found the following features?**<br>Likert scale:<br>1 = Extremely unhelpful<br>2 = Very unhelpful<br>3 = Somewhat unhelpful<br>4 = Neither helpful nor unhelpful<br>5 = Somewhat helpful	Access to the private Facebook group	
		Access to handouts	
		Access to videos from workshops	
		Access to pre-recorded Workshop content (on SharePoint)	
		Workshop Lives (on Teams)	
		Interactive nature of the Workshop Lives	
		Being in a group with other parents/carers	
		Access to lifetime support and advice from the Dogs for Good team	

	6 = Very helpful<br>7 = Extremely helpful		
**15a**	**How important have you found the following features?**<br>Likert scale:<br>1 = Extremely unimportant<br>2 = Very unimportant<br>3 = Somewhat unimportant<br>4 = Neither important nor unimportant<br>5 = Somewhat important<br>6 = Very important<br>7 = Extremely important	Access to the private Facebook group	
		Access to handouts	
		Access to videos	
		Access to pre-recorded Workshop content on SharePoint	
		Interactive nature of the Workshop Lives	
		Being in a group with other parents/carers	
		Access to lifetime support and advice from the Dogs for Good team	
**16**	**Were you (or are you) happy with the mode of delivery?**	Yes, completely	
		Partially, it would have been helpful to have some in-person opportunities	
		I would have preferred in-person workshops	
**16a**	**Please briefly describe why you were (or were not) happy with the mode of delivery.**		
**IN-PERSON WORKSHOPS ONLY**			
**17**	**How helpful have you found the following features?**<br>Likert scale:<br>1 = Extremely unhelpful<br>2 = Very unhelpful<br>3 = Somewhat unhelpful<br>4 = Neither helpful nor unhelpful<br>5 = Somewhat helpful<br>6 = Very helpful<br>7 = Extremely helpful	In-person Workshop presentations at a venue	
		Interactive nature of the in-person Workshops	
		Watching dog training and demonstrations	
		Being in a group with other parents/carers	
		Access to private Facebook group	
		Access to handouts	
		Access to videos	
		Access to lifetime support and advice from the Dogs for Good team	
**17a**	**How important have you found the following features?**<br>Likert scale:	In-person Workshop presentations at a venue	
		Interactive nature of the in-person Workshops	
		Watching dog training and demonstrations	
		Being in a group with other parents/carers	
		Access to private Facebook group	

	1 = Extremely unimportant<br>2 = Very unimportant<br>3 = Somewhat unimportant<br>4 = Neither important nor unimportant<br>5 = Somewhat important<br>6 = Very important<br>7 = Extremely important	Access to handouts	
		Access to videos	
		Access to lifetime support and advice from the Dogs for Good team	
**18**	**Were you happy with the mode of delivery?**	Yes, completely	
		Partially, it would have been helpful to offer both online and in-person opportunities	
		I would have preferred online workshops	
**18a**	**Please briefly describe why you were (or were not) happy with the mode of delivery.**		
**ALL WORKSHOPS**			
**19**	**Was there anything else that you wish had been covered during the workshops you have attended that would have been helpful to you?**	Yes – please specify	
		No	
**20**	**Have you contacted the Dogs for Good team in-between or after the workshops for any advice/support for the following reasons?** *Please select all that apply.*	Dog training advice	
		Dog behaviour advice	
		Advice on selecting the right dog	
		Challenges integrating dog into home	
		Challenges with the bond between your child(ren) and dog	
		Queries around registration status for assistance dogs	
		Signposting to other training/materials	
		Advice on food and nutrition	
		Advice on walking equipment for my child to walk the dog	
		Advice on other equipment for the dog	
		Other (please specify)	
**21**	**Please briefly describe how you think the workshops**	[Free-text]	

	**compared to if you would have attended another conventional training course.**		
**22**	**Would you find any of the following features/content helpful to incorporate into the workshops if you were to complete them again?**	Additional information on integrating puppies into the home (Y/N)	
		Workshops delivered specifically to child(ren) with AUTISM (Y/N)	
		Follow-up module (Y/N)	
		If attended online, breaking Workshop Lives on Teams into smaller modules (Y/N)	
		If attended in-person, breaking three in-person Workshops into shorter sessions over more days (Y/N)	
		Other (Y/N) – If Yes: please specify	
**23**	**Would you recommend the Family Dog Service to others?**	Yes (please specify why)	
		No (please specify why)	
**SECTION E: Impact of your family dog on your child with autism**<br><br>**Please answer the following question thinking about the child your family dog primarily supports. If your family dog supports more than one child with autism, please respond to the questions with just one child in mind.**			
**24**	**How old was your child when you attended the Family Dog Service workshops?**	Rolling age to 17	
**24a**	**How old is your child now?**	Rolling up to 25?	
**24b**	**Which of the following best describes how your child thinks of themselves? My child thinks of themselves as…**	Male	
		Female	
		In another way	
		Prefer not to say	
**25**	**What are/were your main goals and expectations for your family dog?** *Please select all that apply.*	To provide companionship for my child(ren) with autism	
		To promote physical activity	
		To reduce anxiety-based behaviour	
		To have a calming influence on family dynamics	
		To improve my child(ren's) emotional/social skills	

		To improve child(ren's) mental health and wellbeing		
		To promote child(ren's) independence/sense of purpose		
		To promote child(ren's) access to local community		
		Other (please specify)		
**26**	**How was the relationship between your family dog and your child in the <u>first four weeks of your dog's arrival?</u>**	Likert scale:<br>1 = Extremely negative<br>2 = Very negative<br>3 = Somewhat negative<br>4 = Neither negative nor positive<br>5 = Somewhat positive<br>6 = Very positive<br>7 = Extremely positive		
**26a**	**How is the relationship between your family dog and your child <u>at this present time?</u>**	Likert scale:<br>1 = Extremely negative<br>2 = Very negative<br>3 = Somewhat negative<br>4 = Neither negative nor positive<br>5 = Somewhat positive<br>6 = Very positive<br>7 = Extremely positive		
**27**	**Overall, do you believe your family dog has had any positive impact on the following domains for your child?**	Social skills	Y/N	
		Eye contact	Y/N	
		Mood/positive emotions	Y/N	
		Independence/sense of self	Y/N	
		Satisfaction with life	Y/N	
		Self-confidence	Y/N	
		Self-esteem	Y/N	
		Calmness/reduction of agitation and anxiety-based behaviours	Y/N	
		Compliance with demands of daily life (please specify)	Y/N	
		Tolerance in changes in normal routine/increased flexibility in daily routines	Y/N	
		Restrictive/repetitive behaviours	Y/N	
		Tolerance of loud noises	Y/N	
		Tolerance of physical touch	Y/N	
		Level of physical activity, energy and fitness	Y/N	
		Attention, concentration and focus	Y/N	
		Enjoyment with activities and experiences	Y/N	
		Perceptions/feelings about school	Y/N	
		Engagement in new activities or experiences	Y/N	
		Verbal communication	Y/N	

		Non-verbal communication	Y/N	
		Affection/empathy towards others	Y/N	
		Relationships with family members and friends	Y/N	
		Interaction with family members and friends	Y/N	
		Interaction with wider networks/local community	Y/N	
		Sleeping patterns	Y/N	
		Other benefits (please specify)	Y/N	
**28**	**How has your family dog met your goals and expectations for your child?**	Likert Scale<br>1 = Fallen short of all goals/expectations<br>2 = Not at all met goals/expectations<br>3 = Somewhat met goals/expectations<br>4 = Met goals/expectations<br>5 = Exceeded goals/expectations		
**SECTION F: Impact of your family dog on you and your wider family**				
**29**	**How was the relationship between your family dog and you in the first four weeks of your dog's arrival?**	Likert scale:<br>1 = Extremely negative<br>2 = Very negative<br>3 = Somewhat negative<br>4 = Neither negative nor positive<br>5 = Somewhat positive<br>6 = Very positive<br>7 = Extremely positive		
**29a**	**How is the relationship between your family dog and you at this present time?**	Likert scale:<br>1 = Extremely negative<br>2 = Very negative<br>3 = Somewhat negative<br>4 = Neither negative nor positive<br>5 = Somewhat positive<br>6 = Very positive<br>7 = Extremely positive		
**30**	**How was the relationship between your family dog and your other relatives you live with in the first four weeks of your dog's arrival?**	Likert scale:<br>1 = Extremely negative<br>2 = Very negative<br>3 = Somewhat negative<br>4 = Neither negative nor positive<br>5 = Somewhat positive<br>6 = Very positive<br>7 = Extremely positive		
**30a**	**How is the relationship between your family dog and your other relatives**	Likert scale:<br>1 = Extremely negative<br>2 = Very negative<br>3 = Somewhat negative<br>4 = Neither negative nor positive		

	**you live with at this present time?**	5 = Somewhat positive<br>6 = Very positive<br>7 = Extremely positive		
**31**	**Overall, please indicate what impact your family dog has had on you and your family in the following areas:**<br>Likert scale:<br>1 = Extremely negative<br>2 = Very negative<br>3 = Somewhat negative<br>4 = Neither negative nor positive<br>5 = Somewhat positive<br>6 = Very positive<br>7 = Extremely positive	Calm household	1-6	
		Time available for respite	1-6	
		Social support and companionship	1-6	
		Physical activity	1-6	
		Mental health/mood	1-6	
		Satisfaction with life	1-6	
		Flexibility in family daily routines	1-6	
		Interaction with wider networks/local community	1-6	
		Engagement with activities and experiences	1-6	
		Conflict management within the family	1-6	
		Communication within the family	1-6	
		Other (please specify)	1-6	
**31a**	**Have any of these impacts (positive or negative) been unexpected?**	[Free-text]		
**32**	**Please could you provide an estimate of the number of people who have been positively impacted by your family dog?**<br>*This number can include all family members (including those who do not live with you) and those outside the family (e.g., friends).*	[Number insertion only]		
**32a**	**If you have any further comments about how your family dog has impacted these people, please leave them here.**			
**SECTION G: Final comments**				
**33**	**If you have any further comments related to your experience with the Family Dog Service or the impact**	[Free-text]		

	**of your family dog on your family, please leave them here.**		
